# MARK4 protein can explore the active-like conformations in its non-phosphorylated state

**DOI:** 10.1038/s41598-019-49337-0

**Published:** 2019-09-10

**Authors:** Sajjad Ahrari, Fatemeh Khosravi, Ali Osouli, Amirhossein Sakhteman, Alireza Nematollahi, Younes Ghasemi, Amir Savardashtaki

**Affiliations:** 1 Department of Pharmaceutical Biotechnology and Pharmaceutical Sciences Research Center, School of Pharmacy, Shiraz University of Medical Sciences, Shiraz, 71345 15830 Iran; 2 Protein Chemistry Laboratory (PCL), Department of Biology, College of Sciences, Shiraz University, Shiraz, 71964 84334 Iran; 3 Department of Medicinal Chemistry, School of Pharmacy, Shiraz University of Medical Sciences, Shiraz, 71345 15830 Iran; 4 Department of Statistics, College of Sciences, Shiraz University, Shiraz, 71364 84334 Iran; 5 Department of Medical Biotechnology, School of Advanced Medical Sciences and Technologies, Shiraz University of Medical Sciences, Shiraz, 71362 81407 Iran

**Keywords:** Protein structure predictions, Software

## Abstract

Microtubule affinity-regulating kinase 4 (MARK4) is a Ser/Thr protein kinase, best known for its role in phosphorylating microtubule associated proteins, causing their detachment from microtubules. In the current study, the non-phosphorylated conformation of the activation loop was modeled in a structure representing the enzymatically inactive form of this protein, and its dynamics were evaluated through a 100 ns initial all-atom simulation, which was prolonged by another 2 μs. Although the activation loop was folding on itself and was leaning toward ATP site in the initial modeled structure, soon after the initiating the simulation, this loop stretched away from the ATP binding site and stably settled in its new position for the rest of simulation time. A network of hydrogen bonds, mainly between the activation segment residues, αC-helix and the catalytic loop reinforced this conformation. Interestingly, several features of active kinase conformation such as formation of R-spine, Glu106-Lys88 salt-bridge, and DFG-In motif were observed during a considerable number of trajectory frames. However, they were not sustainably established during the simulation time, except for the DFG-In motif. Consequently, this study introduces a stable conformation of the non-phosphorylated form of MARK4 protein with a partially stretched activation loop conformation as well as partial formation of R-spine, closely resembling the active kinase.

## Introduction

MARK4 protein is a Serine/Threonine protein kinase, structurally related to the AMPK/Snf1 subfamily of the CaMK group of kinases^1–3^. MARK4 proteins’ abnormal function has been associated with several pathological conditions including but not limited to carcinomas^4–7^ and Alzheimer’s disease^8–12^.

MARK4 protein consists of N-terminal catalytic domain, common docking motif (CD-like motif), a linker loop, UBA domain, a spacer and the C-terminal tail. The only available crystal structure of MARK4 represents the conformation of kinase core plus UBA domain. In this structure, the human MARK4 protein is in complex with a pyrazolopyrimidine-based small molecule inhibitor^13–15^. Like other MARK isoforms, the enzymatic core is folded into the so-called kinase bilobal structure, with the N-lobe being composed of five β-sheets and holding one α-helix (αC-helix) while the C-lobe comprises of six α-helices and an extended loop, known as the activation segment. This structure is followed by CD-like motif (a sequence of negatively charged residues), linker loop and UBA domain^16^.

The main MARK4 enzymatic activation mechanism is through phosphorylation by LKB1. This upstream kinase phosphorylates Threonine 214 in the T-loop, also known as the activation loop^17,18^. Regardless, MARK proteins have exhibited noticeable basal activity in the non-phosphorylated states of T-loop^17^. Activation loop, as a part of activation segment, starts with the conserved DFG motif and ends at the phosphorylation site (Thr214). Activation segment is further extended by the substrate binding site (P + 1 loop), which is terminated by the APE motif at its C-terminal^19^.

Crystallographic and MD simulation approaches to study the conformations of MARK proteins within the inactive (non-phosphorylated) and active (phosphorylated) states suggest that the activation loop is highly dynamic in the non-phosphorylated kinase structure. However, upon phosphorylation of this loop on the conserved Thr residue (and hence the kinase activation), this loop assumes a stretched conformation which is much more stable in comparison to the non-phosphorylated state^5,15,16,20^.

Although the formation of Lys-Glu salt-bridge was unanimously considered as the hallmark of kinase active structure^19^, recent studies have suggested that stable formation of the R-spine could be a more reliable tag for the active kinase conformation^21–23^. This spine is formed upon the delicate stacking of several hydrophobic residues that are scattered through the N-lobe and C-lobe of the protein^21–23^.

Studies on the conformation transition of kinase proteins have suggested that despite the very flexible nature of activation loop conformation in the non-phosphorylated state, it can visit a plethora of stable conformations, some of which resemble the active and phosphorylated state. In fact, the inactive structures fluctuate in an energy landscapes. The minima of these landscapes could be captured in different and divergent X-ray snapshots^24–26^. These studies suggest that there are two main energy barriers along the path through which the inactive conformation of a kinase switches to its active conformation; the rotation of αC-helix, (which results in the formation of the conserved salt-bridge between a lysine from β3 strand and a glutamate from αC-helix^27–29^) and the T-loop stretching (which cause it to leave the catalytic cleft)^27,28^. During this process, several highly stable conformations can appear in the transition pathway. In these intermediate states, activation segment can adopt an extended conformation, which is prone to phosphorylation by upstream kinases^30–32^.

In this study, we modeled the conformation of activation loop in a crystal structure of MARK4 protein that was introduced as an inactive conformation of MARK4 protein^15^ and evaluated its dynamics through an un-biased MD-simulation approach. Our results indicated that despite the formation of Glu106-Lys88 salt-bridge and R-spine during the simulation time, these spatial motifs are not dynamically stable. Additionally, activation segment assumes a stretched conformation which seems to be competent for the phospho-transfer reaction. In this regard, it seems that during simulation time MARK4 assumes an active-like conformation, which might justify its basal activity in the non-phosphorylated state. The term “non-phosphorylated structure” is used throughout this work to represent the MARK4 original crystal structure, its modeled structure, or the structural products of simulation. The terms “inactive” and “active” represent the kinase structures (crystallographic structures) provided by other teams in the enzymatically inactive and active experimental conditions, respectively.

## Results

### Non-phosphorylated structure model

Table 1 and Supplementary Figure S1 summarize the results on the reliability of the model for the non-phosphorylated MARK4. The results indicated high quality of the modeled structures. The least square fitting of this model on its template also suggested minor structural differences between the modeled structure and the initial template of modeling (with RMSD around 0.02 Å) which suggests conservation of the original crystal structure geometry after model building.

**Table 1:** Model quality for non-phosphorylated-MARK4 structure.

Servers	Feature	Template	Model
Ramachandran Plot	Residues in most favored regions (%)	92	92.1
	Residues in allowed regions (%)	6	6.7
	Residues in disallowed regions (%)	2	1.3
Verify3D	Averaged 3D-1D score >0.2	86.84	90.85
ERRAT	Overall quality	91.55	90.85

### Dynamics of non-phosphorylated conformation of MARK4 through 100 ns simulation

**Figure 1:**
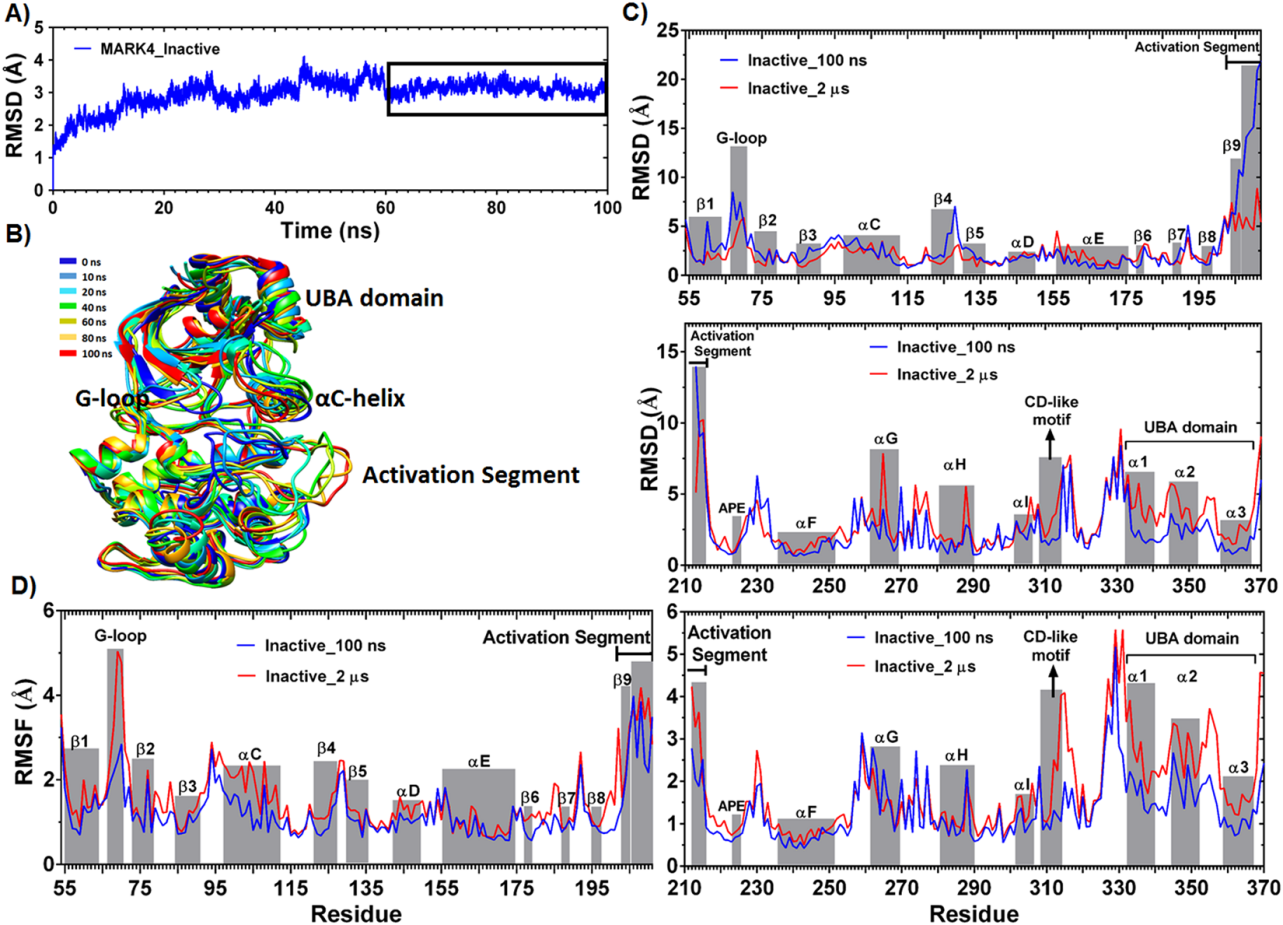
(**A**) Time evolution of the backbone RMSD for non-phosphorylated MARK4 simulated system. (**B**) Six snapshots from different time points of MARK4 non-phosphorylated structure simulation. Color codes represent the extracted structure from each nano-second time point. (**C**) RMSD per residue and (**D**) RMSF per residue are calculated for MARK4 non-phosphorylated structure and during the last 40 ns (blue) and 2 μs (red) of simulation time. Several functionally important motifs and secondary structure elements are tagged in diagrams.

The best model was subjected to 100 ns of all-atom molecular simulation. The backbone RMSD as a function of time reached a relative plateau after about 60 ns of simulation (Fig. 1A). To check the adequacy of the conformational sampling, the production MD period, where the RMSD plot reaches a plateau, was selected, and the cosine content of the first 4 principal components was calculated for this sub-trajectory. The values of the first 4 principal components for this time window were 0.409, 0.370, 0.300, and 0.013, respectively (see Supplementary Fig. S2). These numbers indicate the sufficiency of conformational sampling and convergence of the trajectory.

**Figure 2:**
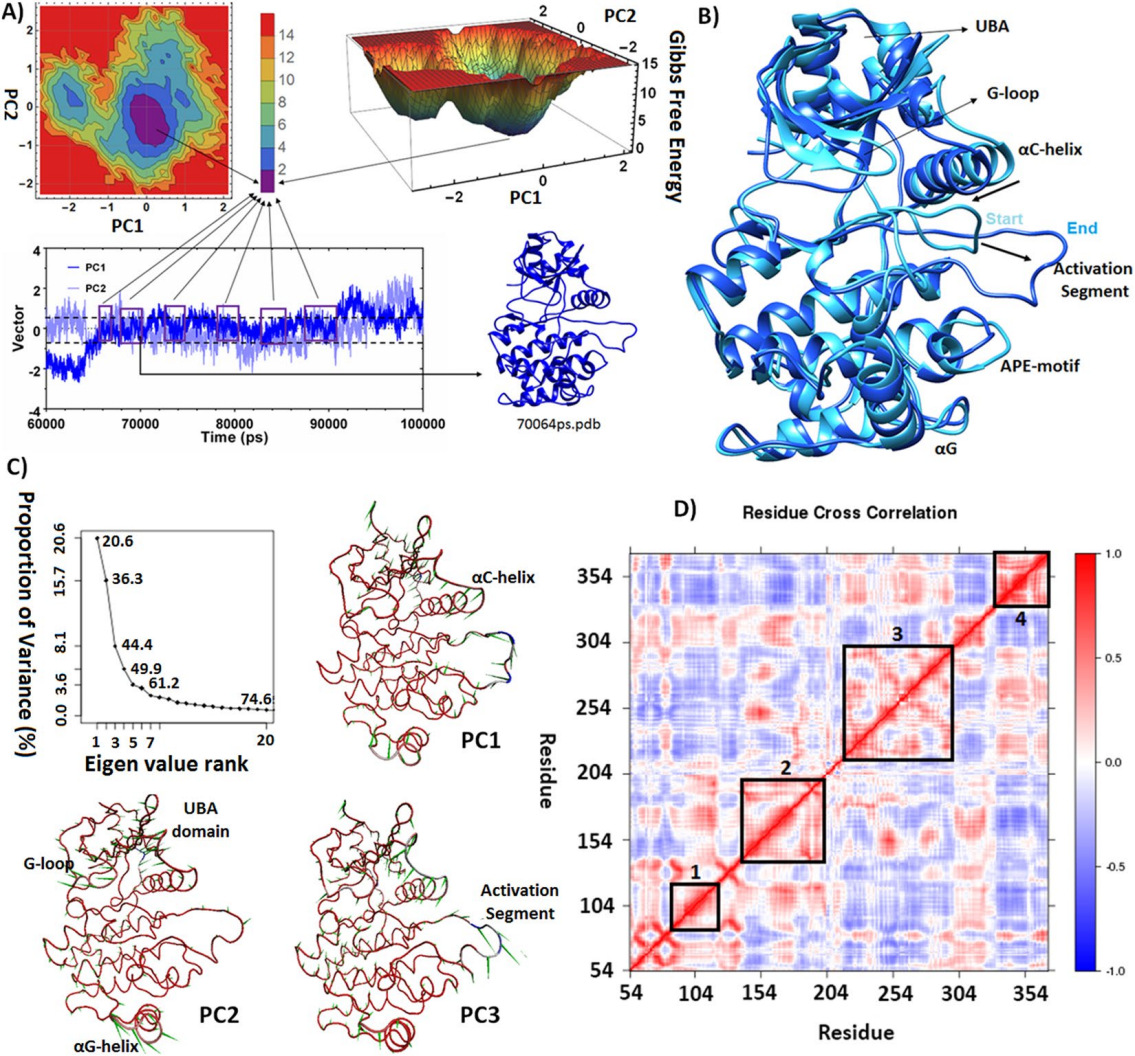
(**A**) 2D and 3D plots of energy landscape built by projections of MD trajectories on two eigenvectors corresponding to the first two PCs. The distribution pattern of the trajectory frames with minimum Gibbs free energy, along the last 40 ns of simulation, is represented, along with the representative MARK4 non-phosphorylated structure extracted from the basin with minimum free energy. (**B**) Least square fitting of representative non-phosphorylated MARK4 structure on the starting structure of simulation. (**C**) Scree plot which represents the proportion of variance for each eigen value of principal component analysis for the last 40 ns of simulation, and porcupine plot which depicts the direction and magnitude of movement of Cα atoms along the first 3 eigen vectors and during the last 40 ns simulation. The direction of the arrow represents the direction of motion. The length of arrow characterizes the strength of the associated movement. (**D**) Dynamic cross correlation map (DCCM) for the last 40 ns of simulation; the X- and Y-axis indicate the Cα residue number and the correlation color is represented in three ranges: blue (anti-correlated and around -1); white (non-correlated and around 0); and red (positively correlated and around + 1). Zones 1–4 are: αC-helix; αE-helix and catalytic loop; activation segment and C-lobe; and finally, UBA domain residues.

Interestingly, during the 100 ns simulation, activation segment went through a stepwise motion resulting in the relatively stretched conformation of this loop. This conformation resembles the active state (Fig. 2B). During the last 40 ns of the simulation, RMSD calculations for each protein residue confirmed that major rearrangements occur in the activation segment, G-loop and the linker loop. However, according to the RMSF plot, both activation loop (205–214) and the N-terminal residues of αC-helix (97–106) were fluctuating during the last 40 ns of simulation (Fig. 2C,D).

In order to extract the representing conformation of MARK4, the special distribution of MARK4 along the first and the second principal components were measured for the last 40 ns of simulation, and the FEL was calculated accordingly. Finally, during this time window a conformation from the basin with minimum free energy was chosen as the representative structure of the non-phosphorylated form of MARK4 protein (Fig. 2A).

The least square fitting of the representative structure with the initial structure of simulation showed that the major structural deviations were associated with G-loop, N-terminal residues of αC-helix and activation segment (Fig. 2B and see Supplementary Table S1). On the other hand, the porcupine plot generated by projecting the trajectory along the first three PCs was in agreement with this pattern. According to this plot, during the last 40 ns of simulation activation loop moves away from the ATP site, while the N-terminal residues of αC-helix approach this zone (Fig. 2C). Additionally, these motions showed a correlated pattern according to DCCM map. Moreover, motions of αC-helix (97–112), catalytic loop (177–186), activation segment (198–226), CD-like motif (312–320) and UBA domain residues (332–370) showed the highest correlation pattern. Since these are the subdomains with major rearrangement during kinase activation, it seems that MARK4 conformation could approach the active structure while it is not phosphorylated on T loop. In addition to this, there was a correlated pattern between the motions of several motifs such as the C-terminal residues of αC-helix (104–112) and motions of β7-β8 strands (187–198) as well as DFG motif (199–202). This pattern was also observed between the motions of activation segment (203–225) and UBA domain (332–370), as well as between the motions of CD-like motif (310–318) and N-lobe of protein (54–138) (Fig. 2D).

### Activation segment conformation upon 100 ns simulation

Upon these motions, activation loop residues reached out for several αC-helix and catalytic loop residues to establish a network of hydrogen bonds. As shown in Table 2, the N-terminal residues of activation segment (Asp199, Phe202, Ser203 and Gly201) are hydrogen bonded to the HRD motif of catalytic loop (His179 and Arg180), or Arg105 from αC-helix. In addition, the C-terminal residues of activation segment, which gives rise to substrate binding site (Lys211, Asp213 and Gly217), are hydrogen bonded to the catalytic loop (Asp181) and the loop following residues of APE motif (Lys231, Tyr232 and Asp233).

**Table 2:** Hydrogen bond map of activation segment residues and the rest of protein during the last 40 ns of first simulation and 2 μs simulation.

Donor		Acceptor		Existence (%)	
Residue	Atom	Residue	atom	100 ns	2 μs
**N-terminal residues**					
ARG105	NH2	SER203	OG	48.958	
LYS88	NZ	ASP199	OD2	29.779	
GLY201	N	HIS179	O	21.639	
GLY201	N	HIS179	ND1	20.999	69.265
ARG180	NH1	GLU205	OE1		51.622
ARG180	NH1	GLU205	OE2		48.453
ARG180	NH2	GLU205	OE2		38.754
ARG180	NH2	GLU205	OE1		34.804
ARG180	NH1	PHE202	O	83.366	54.464
ARG105	NE	SER203	OG	77.941	
PHE202	N	ASP181	OD1		33.145
PHE202	N	ASP181	OD2		38.806
TYR232	OH	ASN204	OD1		76.37
**Substrate binding site (P + 1 loop)**					
SER218	N	ASP181	OD1		48.472
SER218	N	ASP181	OD2		47.016
SER218	OG	ASP181	OD2		21.91
CYS216	N	LEU226	O		21.284
GLY217	N	ASP181	OD2	69.272	
ARG265	NH2	GLY217	O		46.753
ARG265	NH1	CYS216	O		74.214
TYR232	OH	ASP213	O	66.207	
**C-terminal residues**					
SER241	OG	TYR221	O	86.521	89.898
ALA184	N	TYR221	OH		33.458
GLN228	N	PRO224	O		81.641
ARG298	NH2	GLU225	OE1		28.515
PHE227	N	ALA223	O		49.283
LEU226	N	ALA223	O		48.623
ARG298	NH1	GLU225	OE1	99.905	86.176
ARG298	NH2	GLU225	OE2	99.84	87.064
LYS231	N	GLU225	O	83.426	
ALA184	N	TYR221	OH	34.713	
GLY229	N	GLU225	O	24.364	53.369
ARG298	NH2	GLU225	OE1	24.079	
Total				871.00	1263.359

In addition, both the N-terminal residues (199–204) and C-terminal residues (215–225) of activation segment were relatively stable in their location during the last 40 ns of simulation despite the high fluctuation of the middle zone residues (205–214) (Fig. 1D). It seems that this loop is trapped in a relatively stable and stretched conformation, which is exposed to phosphorylation by upstream kinases.

### The Lys88-Glu100 salt-bridge during 100 ns simulation

**Figure 3:**
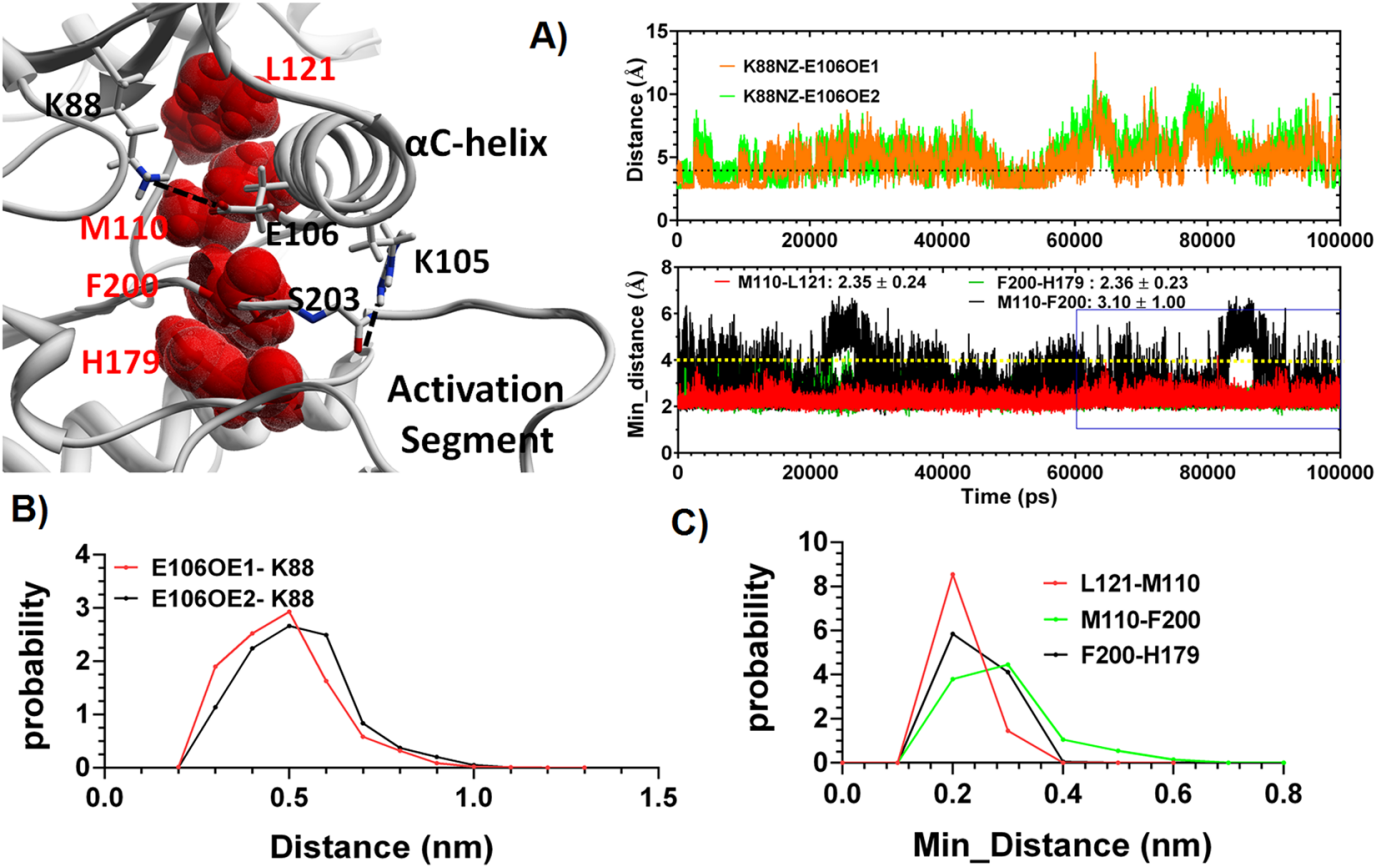
(**A**) The time evolution of distance between Lys88 and Glu106 and the minimum distance between the hydrophobic residues of R-spine. The cut off for the measurement of minimum distance was 4 Å. (**B**) Probability distribution of distance between Lys88 and Glu106 during the simulation time. (**C**) Probability distribution of minimum distance between R-spine residue pairs, during the simulation time.

As the activation loop was stretched, αC-helix was further dragged toward the N-lobe of protein during the simulation. A closer look at αC-helix interactions showed that the conserved Lys88-Glu106 salt-bridge was unexpectedly present within the original crystal of MARK4 at 3.05 Å and was maintained in the representative structure at 3.78 Å. However, during the simulation this salt-bridge was interrupted several times (Fig. 3A). The probability distribution of the distance between the oxygen atom of Glu106 side chain and the nitrogen of Lys88 side chain showed a wide distribution of distances with a pick around 5 Å (Fig. 3B).

### Dynamics of R-spine residues during 100 ns simulation

In addition to activation loop and αC-helix, the dynamics of R-spine residue resembled that of the active structure. This was specifically true in case of Lue121-Met110 and Phe200-His179 residue pairs (Fig. 3A). According to probability distribution plot, the minimum distances between these paired residues explore a narrow range between 1–4 Å with a pick at 2 Å. However, in the case of Met110-Phe200 minimum distance, a wider space was explored, and the distance probability showed an optimum value at about 3 Å, which could be indicative of unstable R-spine formation (Fig. 3C). Since both ATP binding site and substrate binding loop were in a relatively appropriate and stable position to accommodate ATP and substrate, it would be justified to consider this conformation of activation segment as partially active, rather than fully inactive (Fig. 2D).

### Conformation of non-phosphorylated MARK4 through 2 μs simulation

**Figure 4:**
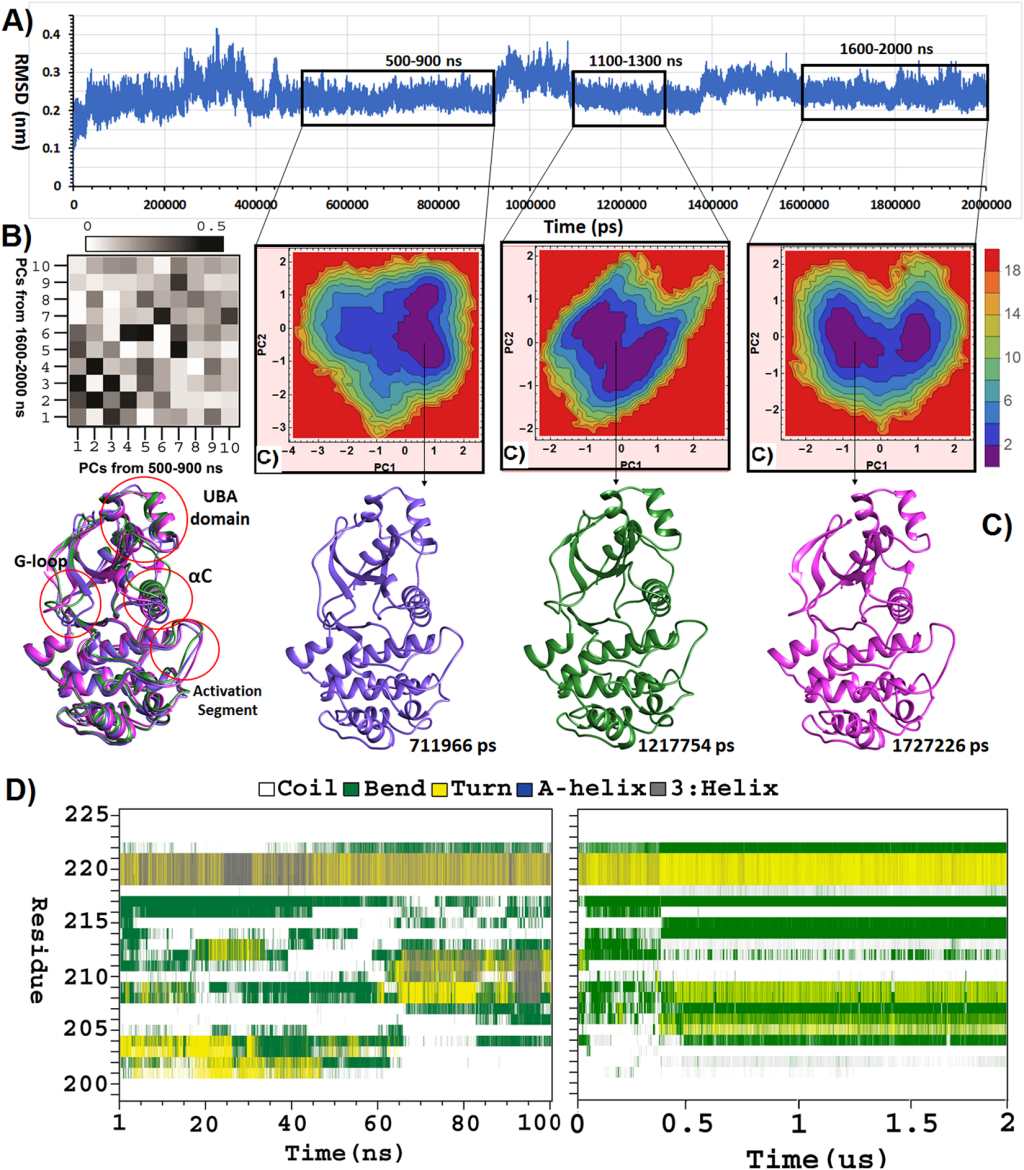
(**A**) Time evolution of the backbone RMSD for non-phosphorylated MARK4 during 2 μs of simulation. (**B**) The RMSIP overlap matrix calculated for the first 10 principal components from the 500–900 ns and 1600–2000 ns time windows of the simulation. The RMSIP values are visualized by the grey gradient from white to black. (**C**) 2D plots of energy landscape built using projections of MD trajectories on two eigenvectors corresponding to the first two PCs. A structure is extracted from the basin with minimum free energy as the representative structure of each time window and the representative structures are overlaid to represent conformational deviations. (**D**) DSSP plot representing the variation of secondary structure for activation segment residues as a function of time, during 100 ns and 2 μs simulations.

In order to evaluate the stability of this conformation, the simulation time was prolonged until 2 μs. As shown in Fig. 4A, RMSD reached a relative plateau with an average RMSD of 2.4 ± 0.2 Å for the last 1600 ns of the simulation. Minor deviations in RMSD suggests for the stable conformation of MARK4 during this time period. To check if this pattern of RMSD was extended beyond 2 μs, the expected pattern was extrapolated up to another 100 ns. To be more precise, the expected pattern is a statistical autoregressive moving average time series ARMA (4, 5), with minimum AICC (A corrected version of the Akaike Information Criteria) equal to −0.778912E + 06 which was fitted on the data. As presented in Supplementary figure S3, the extrapolated RMSD pattern highly resembled that of the last 200 ns, suggesting the maintenance of this pattern if the simulation is further prolonged.

Besides, the convergence of simulations was evaluated through calculating the RMSIP overlap matrix for the ten principal components of the 500–900 ns and the 1600–2000 ns sub-trajectories. The RMSIP matrix showed an overlap among several principal components, with an RMSIP of 0.73, which confirmed the convergence of simulations and the similarity of these two sub-trajectories (Fig. 4B).

The last 1600 ns of simulation was split into three sub-trajectories for further analysis. FEL analysis was performed on these sub-trajectories and the representative structure of each time window was extracted. The least square fitting of these three structures was indicative of negligible differences in term of all-atoms RMSD (0.98 Å). As indicated in Fig. 4C, all three structures represent a stretched conformation of activation segment as that of the non-phosphorylated MARK4 during the initial 100 ns simulation (Figs 2A, 4C).

### Conformational dynamics of activation segment and Lys88-Glu100 salt-bridge upon 2 μs simulation

As the simulation was prolonged, the hydrogen bond network of activation segment with the rest of protein was strengthened (Table 2). This pattern was particularly distinctive for the N-terminal residues of the activation segment as well as the P + 1 loop residues. According to this pattern, the N-terminal residues of activation segment, that cover the residues of DFG motif, were mainly tethered to the residues of αC-helix, and catalytic loop. Interestingly, Asn204 and Glu205 made a huge contribution to the formation of this network, as the simulation was extended to 2 μs. On the other hand, the P + 1 loop residues were hydrogen bonded to the residues of αG-helix or the loop following the APE motif (Table 2).

**Figure 5:**
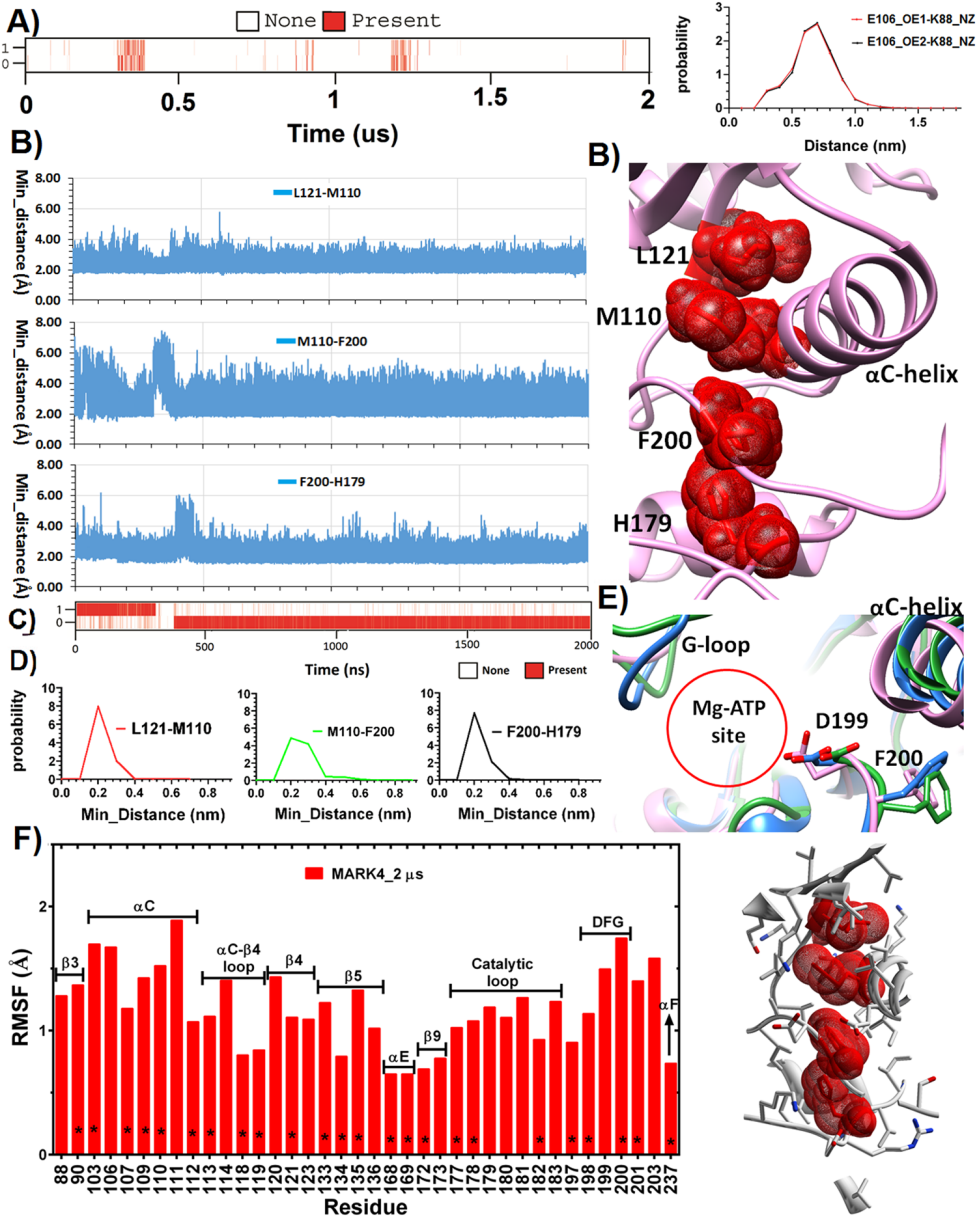
(**A**) Time evolution of hydrogen bonds between Lys88 and Glu106 and the probability distribution of distances between the atoms engaged in salt-bridge formation. (**B**) Time evolution of minimum distance between residue pairs of R-spine. The cut-off for the measurement of minimum distance was 4 Å. (**C**) Time evolution of hydrogen bonds between Asp238 side-chain and His179 backbone. (**D**) Probability distribution of minimum distance between residue pairs of R-spine. (**E**) Least square fitting of MARK4 crystal structure (green) on the representative structure of 100 ns simulation (blue) and the representative structure of the last 400 ns of the second simulation (pink). The conformation of DFG motif residues is highlighted. (**F**) RMSF of residues with minimum distance of ≤4Å from R-spine residues during the 2 μs simulation.

As shown in Fig. 1D, both the N-terminal residues of activation segment (199–203) as well as the residues of P + 1 loop (215–220) have a relatively low RMSF during this 2 μs simulation, suggesting for a stretched and stable conformation of P + 1 loop which might be able to accommodate the substrate. Furthermore, the secondary structure of activation segment residues was relatively stable during the last 1600 ns of simulation (Fig. 4D). Despite this, the Glu106-Lys88 salt-bridge was broken upon the second simulation. According to the probability distribution plot, the distance between corresponding atoms of Glu106 and Lys88 explored a wide range and showed a peak at around 6 Å (Fig. 5A).

### Dynamics of R-spine residues during 2 μs simulation

The minimum distance between the hydrophobic residues of R-spine was indicative of partial formation of this spine during the 2 μs simulation time. Additionally, the hydrogen bond between Asp238 side-chain and His179 backbone was stably formed during the last 1600 ns of simulation (Fig. 5B,C). As shown in Fig. 5D, the minimum distance between Leu121-Met110 and Phe200-His179 had a narrow probability distribution of around 2 Å. Whilst, in the case of Met100-Phe200, the probability of minimum distance explored a wider window as well as a lower probability at 2 Å. Despite the formation of R-spine in the majority of the frames, the wobbling motions of αC-helix seems to disturb the sustained formation of the R-spine. Interestingly, both in the initial crystal structure and in the representative structures of conformational sampling during the 2 μs simulation, MARK4 represented a DFG-In conformation, which is odd for an inactive kinase structure (Fig. 5E).

As shown in Fig. 5F, R-spine residues were surrounded with a stable array of hydrophobic residues, and were highly shielded from solvent molecules (the solvent accessible surface area (SASA) for Lue121, Met110, Phe200 and His179 were 9.3 ± 5.0 Å^2^, 9.4 ± 7.0 Å^2^, 13.2 ± 9.8 Å^2^ and 6.4 ± 6.6 Å^2^ respectively). This hydrophobic cage could further reinforce the formation of R spine. There are seven missense SNPs at the location of residues surrounding the R-spine. The predicted deleterious effects of these mutations are summarized in Table 3.

**Table 3:** Prediction results of Deleterious SNPs in MARK4 gene using eleven web servers.

Mutation	K88E	I109V	I118V	V169M	I177V	D181E	I197T
Server							
Polyphen2	D	N	D	D	N	D	D
PhD-SNP	D	N	N	D	N	D	D
SIFT	D	D	D	D	N	D	D
Meta-SNP	D	N	N	N	N	D	D
PANTHER	—	N	N	D	N	D	D
Mutationassessor	High	Neutral	Low	Low	Low	High	Medium
SNAP	D	N	N	D	N	D	D
PROVEAN	D	N	N	D	N	D	D
SNPs&GO	D	N	D	D	N	D	D
NetDiseaseSNP 1.0	D	N	N	D	N	N	D
Align GVGD	ClassC55	ClassC25	ClassC25	ClassC15	ClassC25	ClassC35	ClassC65

D = Disease, N = Neutral.

Three SNPs including K88E (rs57927646), D181E (rs777001749) and I197T (rs1010263943) had the highest deleterious effects. Moreover, these three SNPs were predicted to reduce protein stability (see Supplementary Table S2). The effect of K88E and D181E mutations could be justified regarding their role in the formation of K88-E106 salt-bridge and localizing the Mg-ATP complex, respectively. However, for the I197T mutation, the effects could be attributed to destabilizing the R-spine formation or interfering with the communication of αC-β4 loop and C-lobe of protein. This residue fills a hydrophobic pocket beneath the αC-β4 loop and can mediate the communication of this loop with C-lobe of MARK4 (see Supplementary Fig. S4).

## Discussion

In this study, we modeled the conformation of activation loop in a crystal structure of non-phosphorylated MARK4 protein (PDB ID: 5ES1)^15^ and studied the kinase core dynamics in the presence of the modeled loop.

The original crystal structure was introduced as an inactive form of the protein, regarding the conformation of activation segment^15^. However, this structure holds several features of active kinase conformation which worth investigating it through a dynamic perspective; First, αC-helix assumes a closed conformation and is packed against the kinase N-lobe and permits the formation of the conserved Lys-Glu salt-bridge. Formation of this salt-bridge is considered to be the hallmark of kinase activation. Moreover, the spatial orientation of R-spine residues highly resembles that of the active kinase conformation, and the lease-square fitting of this structure on the active conformation of MARK2 protein suggests for minor structural deviations (with an RMSD of 0.7 Å). Despite these active-like conformational features, the activation loop is not phosphorylated (according to the methodology of crystallography^15^). Experimental studies have suggested that full activation of MARK4 relies on the phosphorylation of the conserved Thr214 residue on the activation loop^17^. Based on this phosphorylation, the activation loop assumes a stretched and stable conformation, none of which could be inferred from the crystal structure. In fact, most of the activation loop residues are missed from the crystal structure and they are highly fluctuating according to their B-factor in this crystal structure (PDB ID; 5ES1).

Based on these features, kinase could be regarded as enzymatically inactive in this structure (due to lack of phosphorylated Thr214; hence, a radical fluctuation in activation loop which does not allow for the stable stand of the substrate on the P + 1 loop). Meanwhile, this 3D structure holds the hallmark features of an active kinase.

To have a better understanding of the enzyme behavior in this condition, we patched the missing resides of the activation loop through a rigorous modeling approach and with 10000 modeling trials and inspected the dynamics of the modeled structure through 2 μs simulation.

Our simulation results are at odds with conventional descriptions of a kinase conformation in its enzymatically inactive and active states. This static description, which was mostly developed based on the results of crystallographic approaches, describes the structure of the enzymatically active form of protein by holding several features such as the stacked R-spine, established Lys-Glu salt bridge and DFG-In motif^19^. In the recent years, several MD simulation studies have provided a dynamic perspective of the structures pertaining to inactive and active states of kinase proteins. This new model suggests that the conformation of the enzymatically inactive kinase can explore a wide and various landscape, with several more stable and energetically favored minima. In this regard, the inactive conformations that sit in these minima may assume features that are associated with the active conformation, but the stability of such active-like features and the probability of their exploration is varied between different kinases. Additionally, the phosphorylation of activation loop contributes to the kinase activation by stabilizing these features as well as promoting the likelihood of their establishment^25,26,33^. In fact, after the T-loop phosphorylation, the phosphate moiety mediates the communication of the two lobes of the kinase core and facilitates the assembly of the R spine, which leads to stabilizing the active conformation^34^. This active conformation can now perform the so-called breathing motion and locally switch between the open and closed states that are associated with catalysis^35^. This model justifies the observation in which active structures are similar between different kinase portions, while each kinase can have its own fashion of being inactive^36^. We observed that despite the high flexibility of the activation loop, the non-phosphorylated conformation of MARK4 it is stably trapped in one or two low energy bins with activation segment assuming a stretched and relatively stable conformation, which is reminiscent of the active conformation. In this active-like conformation, features such as DFG-In motif allow for both the ATP localization as well as the substrate stable stand on the P + 1 loop. In a study by Meng *et al*., a similar behavior of activation loop was observed for the c-Src kinase^25^. Their findings are also indicative of R-spine establishment within the non-phosphorylated state of T-loop. However, according to their PMF analysis, R-spine conformation explores two minima in the non-phosphorylated state, which is indicative of lower stability compared to one minima energy landscape in case of the phosphorylated state^25^. Our results also suggest that the residues of the R-spine are stacking on top of each other during a noticeable number of simulation frames. However, the distance between M110-F200 is highly fluctuating and does not permit for a stable R-spine formation (Fig. 5B).

The stable formation of the conserved Glu-Lys salt-bridge has been suggested as the major energy barrier in the path through which the Src kinase switches from inactive to active conformation^25,26^. Therefore, the sustained formation of this slat-bridge would be less likely in the non-phosphorylated active-like conformation. This is in agreement with our observation for the Glu100-Lys82 salt-bridge formation throughout the simulation, which has a flickering pattern along the 2µs simulation. Despite this, establishment of activation loop and R-spine in the active-like conformation could permit for the phosphor-transfer reaction and hence the basal enzyme activity of MARK4 in this conformation^17^. However, the stable formation of Lys88-Glu100 salt-bridge is indispensable for the full enzymatic activity. It should be noted that upon T-loop phosphorylation and RD-pocket formation, C-helix is stably tethered into the phosphate moiety in it so called “In” conformation^20,21^. This conformation would be further stabilized by Lys88-Glu100 salt-bridge formation and allow for the stable communication of R-spine resides and Met110-Phe200, in particular (Fig. 5B). In other words, it seems that we are dealing with an active-like conformation of MARK4 protein which has overcome the first barrier along the activation path (T-loop stretching) yet it is not fully active as it has not passed the second barrier (stable formation of Glu-Lys salt-bridge).

The presence of an active-like conformation in MARK4 protein could be attributed to several features.

First, the existence of an ample hydrophobic shell around the R-spine residues may facilitate the R-spine stacking in the non-phosphorylated form of the protein;

As reviewed by Kornev *et al*.^23^, a conserved array of hydrophobic residues surrounding the R-spine can affect the feasibility and stability of R-spine formation; hence, the kinase activation. In case of MARK4 protein, an ample hydrophobic shell surrounds the R-spine residues and shields them from the solvent molecules. This could reinforce the formation of this spine during the non-phosphorylated state and explain the basal activity of MARK proteins in the non-phosphorylated states of T-loop^17^. Consequently, existence of such intermediate conformation which is cocked for activation (according to R-spine and activation segment localization), would explain the dramatic increase in MARK4 activity upon the induction of T214E mutation^17^. In several kinase proteins, the mutations of the hydrophobic residues around R-spine have been associated with dramatic changes in kinase functionality^23^. Although none of the mutations in the corresponding residues of MARK4 were reported to change the kinase activity, the I197T mutation (which belongs to this hydrophobic cage and is located on the loop preceding the DFG motif), was predicted to have a deleterious effect on MARK4 stability and function. This residue fills a pocket underneath the αC-β4 loop, and its motions shows a correlated pattern with that of αC-β4 loop (Fig. 2D). Such amino acids, that mediate the communication of αC-β4 loop and the C-lobe of protein, have been suggested to have an important role in the protein functionality^23^. The importance of this hydrophobic shell is more evident regarding its conservation among the members of AMPK subfamily (see Supplementary Fig. S5). Interestingly, MELK protein, that has the highest basal kinase activity compared to other members of AMPK subfamily^17^, also has a higher hydrophobic index for this shell of residues (Fig. 5F and see Supplementary Fig. S5). Despite the partial formation of R-spine, phosphorylation of T-loop seems to be indispensable for the fulfillment of a stable active conformation. It seems that upon phosphorylation, the positively charged N-terminal residues of αC-helix are tethered to the phosphate moiety and restrain the wobbling motions of αC-helix, which can otherwise interfere with Glu-Lys salt-bridge formation.

Second, the unexpected presence of Asn and Glu residues on the β9 strand (Asn204 and Glu205 in MARK4), (which was discussed in our previous study^20^);

It was suggested that activation of RD kinases that bear negatively charged or neutral amino acids on this location, is exempt from T-loop phosphorylation^29,37^. This is probably due to the ability of these residues to mimic the phosphate moiety in formation of the RD pocket and gathering the basic residues of αC-helix and catalytic loop. Intriguingly, according to Table 2, Glu205 and Arg180 (from catalytic loop) established a strong network of hydrogen bond with αC-helix and catalytic loop, which stabilizes the stretched conformation of activation loop and might facilitate the formation of RD pocket in the absence of phosphate group.

Third, the existence of a DFG-In motif in the non-phosphorylated state of MARK;

The existence of DFG-In motif for an inactive kinase conformation is not expected. However, this motif was preserved both in the original crystal structure of MARK4 and trajectory products of MD simulation. It is possible that existence of an inhibitor within the ATP site has molded the MARK4 conformation to adopt this odd orientation. However, our previous study on the active and inactive conformations of MARK2 protein suggested for the existence of this conformation in both inactive and active structures of MARK2 protein, as well^20^. The existence of this DFG-In motif, along with the hydrophobic shell around R-spine residues and the existence of negatively charged residues on β9 strand might permit the kinase to explore the active-like conformations while not being phosphorylated and facilitated the formation of R-spine within the non-phosphorylated state.

## Conclusion

In this study, we modeled the conformation of the activation loop in a crystal structure of MARK4 protein, which was originally introduced as an inactive structure of MARK4 protein. However, our molecular modeling approach is suggestive of an active-like structure for MARK4 protein in terms of activation segment and αC-helix conformations as well as the dynamics of R-spine residues.

Along the simulation time, the activation loop leaves the ATP binding site and assumes a stretched conformation. This conformation is stabilized through a network of interactions with αC-helix and catalytic loop. This conformation may allow the ATP and substrate proper localization. Additionally, the conservation of DFG-In motif may further facilitate the ATP positioning and the phosphor-transfer reaction. However, the wobbling motions of the αC-helix does not permit for the sustained formation of the conserved Glu100-Lys82 salt-bridge and despite the establishment of this salt bridge in the original crystal structure of MARK4, it was dissociated upon the elongation of simulation time. Moreover, this wobbling motions of αC-helix does not permit the delicate stacking between Met110 and Phe200 which results in the flickering pattern of R-spine stacking.

Despite this, it seems that the non-phosphorylated MARK4 protein can assume active-like conformations (that might be competent for enzymatic reaction) in a noticeable number of trajectory frames. This observation presents a structural clue for the basal activity of protein in the non-phosphorylated state. We speculate that the formation of a stable active-like conformation in MARK4 protein could be either due to the ample hydrophobic shell around the R-spine (which shields the R-spine and facilitate its formation) or the unexpected presence of DFG-In motif in MARK4 non-phosphorylated structure.

The unique features of this active-like conformations, such as the formation of DFG-In motif, could be exploited for drug design purposes to minimize the off-target interactions.

## Methods

### Modeling

The geometric coordinates of a human derived MARK4 protein was used as the main template for building the non-phosphorylated structures (PDB IDs: 5ES1) using MODELLER software version 9.16^38,39^. This crystal structure represents the inactive form of protein in complex with a pyrazolopyrimidine-based inhibitor and covers the residues of kinase core, CD-like motif and UBA domain (54–370)^15^. As expected from an inactive kinase structure, the majority of activation loop residues (205–218) are missing from the crystal, due to high fluctuations. Since the position of this loop is highly varied among different kinases, no extra templates were used in the reconstruction process, and the missing loop was modelled based on ab-initio/loop refinement method using the loop modeling function of MODELLER^38^. Out of 10,000 loop refinement trials, the one corresponding to the lowest value of the energy and Dope score was selected and renumbered according to the canonical sequence of human MARK4 protein (UniProt ID: Q96L34). The inhibitor’s structure was stripped from the complex to yield the starting structure of simulation. To check the quality of the model, ERRAT^40^ and VERIFY3D^41^ software packages, as well as the RAMPAGE web server^42^ were used.

### MD simulation

All simulations were performed using GROMACS v.5.1^43^ with CHARMM36 force field parameters^44^. The starting structure of non-phosphorylated MARK4 structure, as prepared by MODELLER, was centered in a cubic box and then immersed in TIP3P water molecules. The minimum distance between the protein and the box boundaries was 1.0 nm. The system net charge was neutralized by replacing water molecules with Cl^−^ ions. The system was energy-minimized using the steepest descent algorithm until the maximum force on each atom was smaller than 1000 kJ/mol.nm. After energy minimization, the temperature and pressure of the system were calibrated by two separate position-restrained MD simulations; to adjust the temperature, an NVT MD simulation was performed for 100 picoseconds (ps) at 300 K using the velocity rescale algorithm with τT = 0.1 ps^45^. After reaching the correct temperature, NPT MD simulation at 300 K and 1 bar for 200 ps was performed by the Parrinello-Rahman algorithm with τP = 2.0 ps during which density of the system was stabilized at around 1000 kg/m3^46^. The integration step for all simulations was 2 fs and the interval for data collection was set to 2 ps. The Particle Mesh Ewald (PME) algorithm was applied to calculate long-range electrostatics interactions with a cutoff of 1.2 nm, and a cutoff of 1.2 nm was set for Van der Waals interactions^47^. The Verlet integrator with an integration time step of 2 fs was used and LINCS algorithm was employed to keep all bonds involving hydrogen atoms rigid^48^. Each system component was coupled separately to a thermal bath (protein was considered as one component and water plus ions were considered as the other component), and isotropic pressure coupling was used to maintain the pressure at the desired value. Finally, the MD simulation for 100 nanoseconds (ns) at constant pressure and temperature was performed. The simulation was prolonged by 2 μs with the same protocol and using the last frame of the previous simulation as the starting structure. Each simulation started from a different conformation with a different random seed. The secondary structure database (DSSP) was installed into GROMACS to analyze protein secondary structure changes^49^, and structural diagrams were prepared by UCSF Chimera 1.11 software^50^.

### Principal component analysis (PCA)

PCA was performed using GROMACS v.5.1 tools^43^. The “gmx covar” function was used to yield the eigenvalues and eigenvectors by calculating and diagonalizing the covariance matrix, whereas the “gmx anaeig” tool was used to analyze the eigenvectors. The eigenvalues were obtained by the diagonalization of the covariance matrix of the Cα atomic fluctuations. Bio3d package of R software, which was specifically developed to analyze bio-molecular data^51,52^ was used for representing the scree plot of principal component analysis and the 2-Dimensional projection of eigenvectors. Only the atomic coordinates of the Cα atoms were used in the analysis. To represent the movement directions captured by the eigenvectors, the porcupine plot was generated using 30 extreme projections on principal components PC1, PC2 and PC3 as the input for the Prody plugin of VMD software^53,54^. The arrow direction in each Cα atom represents the direction of motion, while the length of arrow characterizes the strength of the associated movement.

The statistical significance of convergence of the trajectories was obtained by calculating the cosine content of the first 4 principal components, as well as the root-mean-square inner product (RMSIP) over the first 10 eigenvectors of the Cα atoms^55,56^. It has been shown that when the first principal component is similar to a cosine with half a period, the sampling is far from converged^57^. Hence, the lower this cosine similarity is, the higher the chance of conformational sampling convergence. On the other hand, RMSIP values range from 0 to 1; the value is 1 if the sampled subspaces are identical and 0 if they are orthogonal. Values of RMSIP ≥0.6 are considered good convergence while RMSIPs ≥0.8 are considered excellent^55^.

### Cross-correlation analysis

Correlated atomic motion in the non-phosphorylated MARK4 structure was obtained by analyzing the dynamical cross-correlation map (DCCM) using the bio3d package of R^51,52^. Only the atomic coordinates of the Cα atoms were used in the analysis to reduce statistical noise, and to avoid apparent correlations between slow side-chain fluctuations and backbone motions. The last 40 ns of the first simulation was used for this analysis.

### Free energy landscape

The calculation of the free energy landscape (FEL) was performed using the gmx sham utility of GROMACS and according to the following Eq. (1)^58^;

1 $${G}_{i}=-\,{k}_{B}T\,\mathrm{ln}(\frac{{N}_{i}}{{N}_{\max }})$$

where *k*_*B*_ is Boltzmann’s constant, T is the temperature of simulation systems. *Ni* is bin i population and *Nmax* is the population of the most populated bin. Bins with no population are given an artificial barrier scaled as the lowest probability. FELs were represented using the first two principal components of the system, as a measure of conformational variability. Free energy was estimated from populations (probability distributions) of the system with respect to the chosen variables. When represented in three dimensions, the landscape shows ‘valleys’ of low free-energy, which represent metastable conformational states of the system, and ‘hills’ that account for the energetic barriers that connect these states. Different energy levels are displayed using color-code modes. The trial version of Mathematica software was used to represent the 2D and 3D FEL graphs (Wolfram Research, Inc., Champaign, IL, USA, 2018). Several structures were extracted from the low-energy region of the plot using “get_timestamp.py” script.

### Disease-associated SNP prediction

Information about SNPs of MARK4 gene was collected from the NCBI database (https://www.ncbi.nlm.nih.gov/snp). Then, eleven web-based bioinformatics servers were used to evaluate the deleterious effects of the selected SNPs from the previous step. These servers included Polyphen2^59^, PhD-SNP^60^, SIFT^61^, Meta-SNP^62^, PANTHER^63^, Mutationassessor^64^, SNAP^65^, PROVEAN^66^, SNPs&GO^67^, NetDiseaseSNP 1.0^68^, Align GVGD^69^. In addition, the effect of these SNPs on the stability of the protein was investigated with the I-Mutant 3.0 server^70^. Information about 12 web-based servers used in this article is summarized in Supplementary Table S3.

### Data extrapolation (Prediction)

The expected pattern is the following autoregressive moving average time series model based on the standard Box and Jenkins method)^71^:

2 $$\begin{array}{rcl}X(t) & = & {\rm{0.7312}}X(t-{\rm{1}})+{\rm{0.5792}}X(t-{\rm{2}})\\  &  & +{\rm{0.2770}}X(t-{\rm{3}})-{\rm{0.5887}}X(t-{\rm{4}})\\  &  & +Z(t)+{\rm{0.06176}}Z(t-{\rm{1}})-{\rm{0.4809}}Z(t-{\rm{2}})\\  &  & -{\rm{0.6311}}Z(t-{\rm{3}})+{\rm{0.09060}}Z(t-{\rm{4}})+{\rm{0.03857}}Z(t-{\rm{5}})\end{array}$$

This Eq. (2) is an ARMA (4, 5) model, where *X*(*t*) is the given time series at time *t*, and Z(*t*) is the white noise process. The statistical software R^52^ was applied to fit the best model. The minimum AICC (a corrected version of the Akaike Information Criteria) was equal to −0.778912E + 06 which confirms the model validity. The model is then used to extrapolate the RMSD fluctuations for another 100 ns (beyond the 2 μs period), and based on the RMSD values for last 200 ns of simulation.

## Supplementary information

Supplementary Information

## Data Availability

The materials, data and associated protocols will be promptly available to readers upon the request.
