# Supplementary material for: MARK4 protein can explore the active-like conformations in its non-phosphorylated state
Source: Sci Rep. 2019 Sep 10;9:12967. doi: 10.1038/s41598-019-49337-0 (PMC6737064; doi:10.1038/s41598-019-49337-0)
Supplement: Supplementary file 1 — Supplementary Information [file 41598_2019_49337_MOESM1_ESM.doc]

**MARK4 protein can explore the active-like conformations in its non-phosphorylated state**

Sajjad Ahrari^1^, Fatemeh Khosravi^2^, Ali Osouli^1^, Amirhossein Sakhteman^3^, Alireza Nematollahi^4^, Younes Ghasemi^1^, Amir Savardashtaki^1,5^,*.

^1^ *Department of Pharmaceutical Biotechnology and Pharmaceutical Sciences Research Center, School of Pharmacy, Shiraz University of Medical Sciences, Shiraz 71345 15830, Iran.*

*^2^ Protein Chemistry Laboratory (PCL), Department of Biology, College of Sciences, Shiraz University, Shiraz 71964 84334, Iran.*

*^3^ Department of Medicinal Chemistry, School of Pharmacy, Shiraz University of Medical Sciences, Shiraz 71345 15830, Iran.*

*^4^ Department of Statistics, College of Sciences, Shiraz University, Shiraz 71364 84334, Iran.*

*^5^ Department of Medical Biotechnology, School of Advanced Medical Sciences and Technologies, Shiraz University of Medical Sciences, Shiraz 71362 81407, Iran.*

*Corresponding author. Tel.: 987136281407; Fax: 987136281506

E-mail address: dashtaki@sums.ac.ir


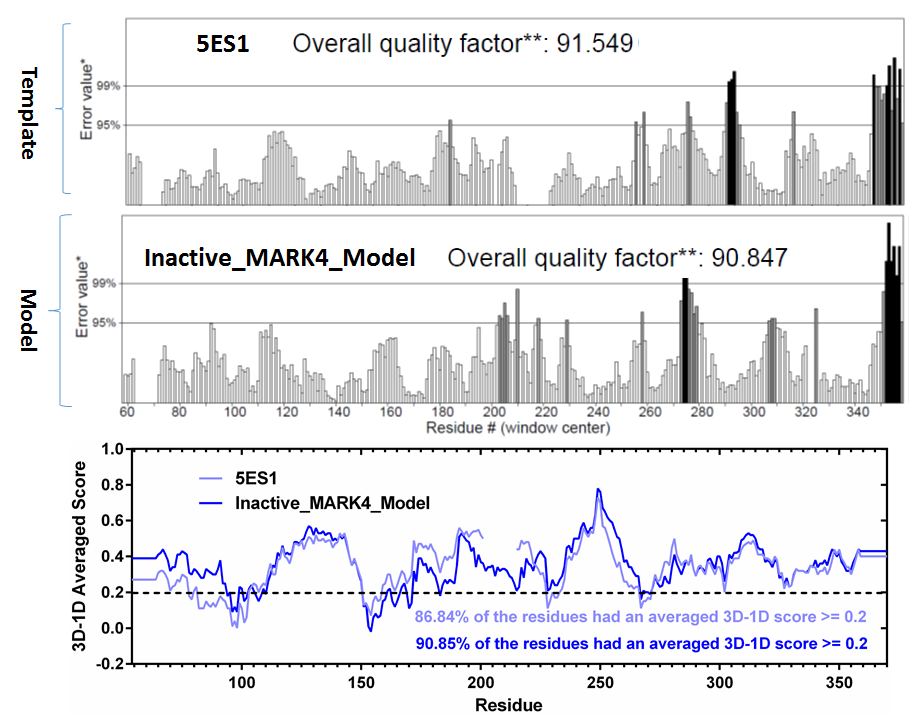


Supplementary Fig. S1. Result of ERRAT and VERIFY-3D analysis for the model and its template. The PDB IDs of inactive templates is presented.


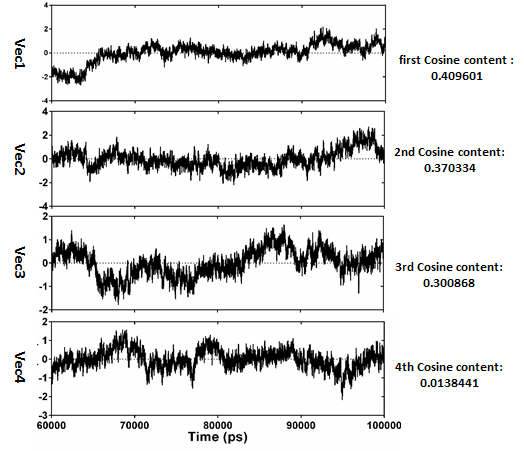


Supplementary Fig. S2. Cosine content for the projection of the last 40 ns trajectory, along the first 4 Eigen Vectors.


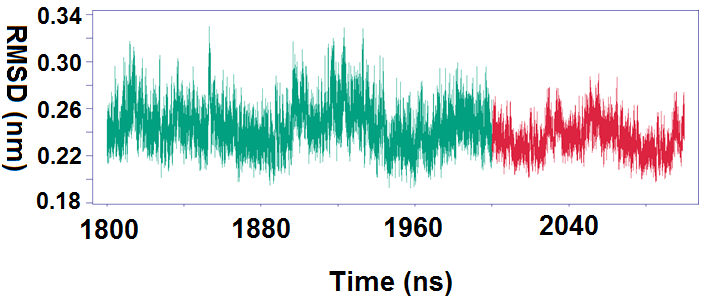


Supplementary Fig. S3. Prediction of RMSD pattern beyond the 2μs time point.


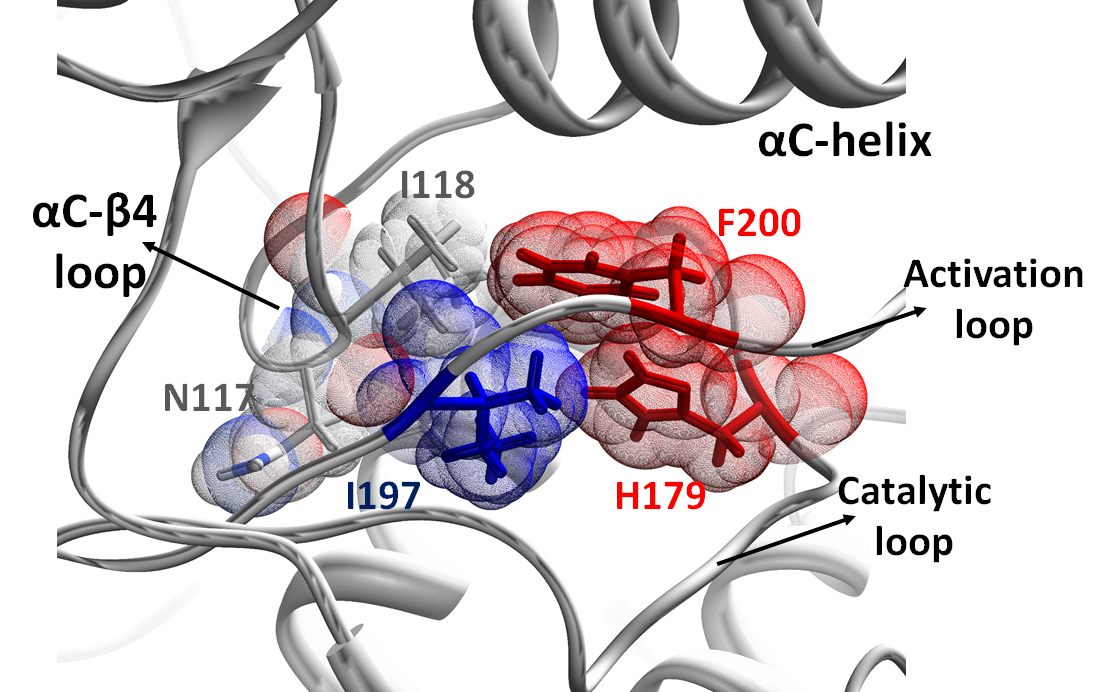


Supplementary Fig. S4. Interaction of Ile197 with αC-β4 loop and R-spine residues.


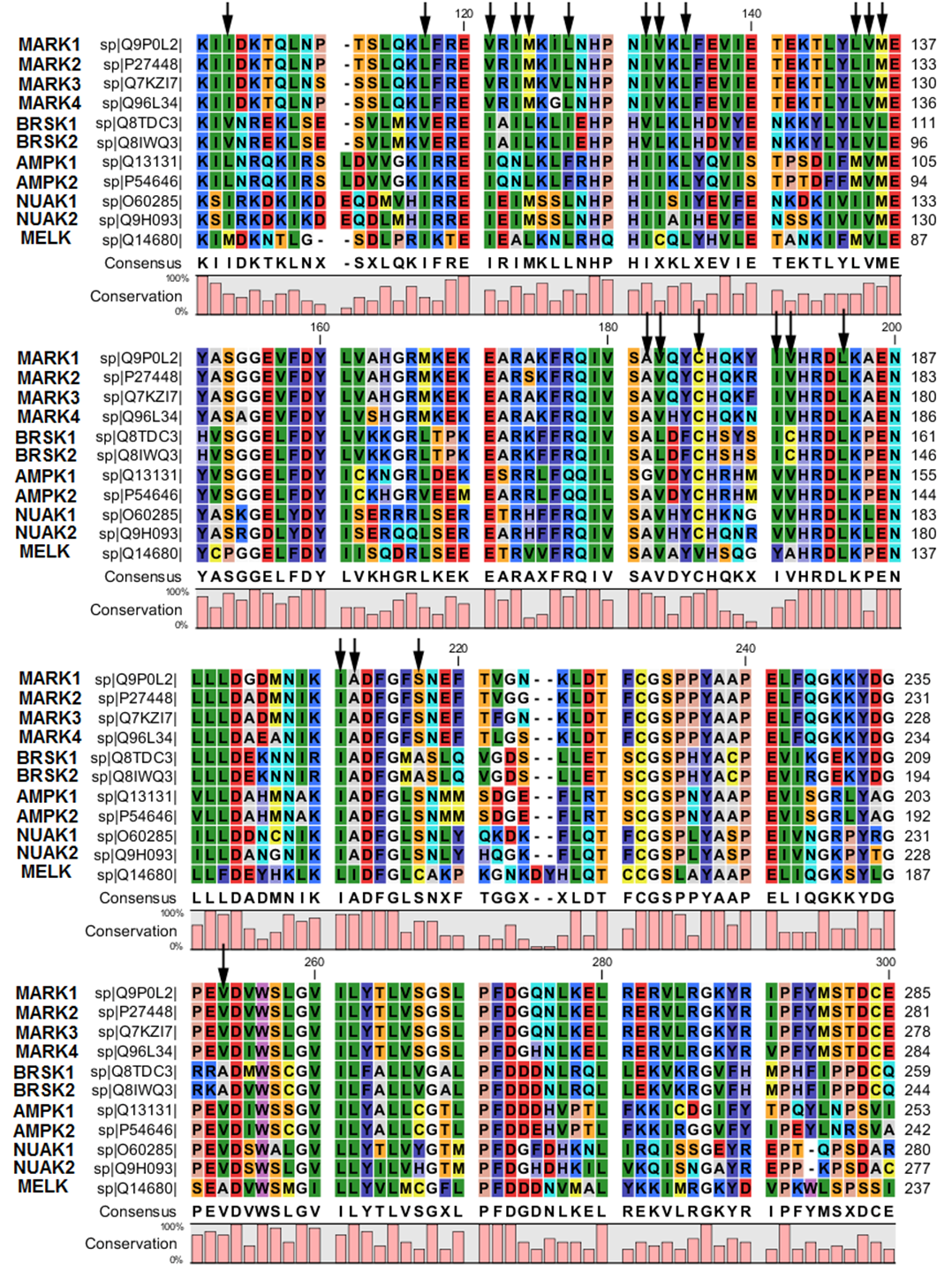


Supplementary Fig. S5. The residues of hydrophobic shell around R-spine in the members of AMPK subfamily. These residues are tagged with the vertical arrows.

| **Subdomain (residues)** | N-lobe  (54-137) | G-loop  (66-72) | αC-helix  (96-112) | Activation segment  (193-219) | Linker + UBA  (305-363) |
| --- | --- | --- | --- | --- | --- |
| **RMSD (Å)** | **2.67** | **3.22** | **2.62** | **26.37** | **2.51** |

**Supplementary Table S1. Backbone RMSD of the protein residues after the least square fitting of MARK4 model and the representative structure of 100 ns simulation.**

| **Amino acid (AA) change** | **Stability** | **DDG Value Prediction** |
| --- | --- | --- |
| **K88E** | Decrease | -0.48    Kcal/mol |
| **D181E** | Decrease | -0.36    Kcal/mol |
| **I197T** | Decrease | -2.11    Kcal/mol |

**Supplementary Table S2. Prediction result of stability of protein structure by I-Mutant server.**

**DDG (delta delta G) represents free energy change**

| **Web servers** | **Input** | **Output**  **Prediction** | **Ref** |
| --- | --- | --- | --- |
| **Polyphen2**  http://genetics.bwh.harvard.edu/pph2/ | Protein Sequence | Score ranges from 0 to a positive number, where 0 is neutral, and a high positive number is damaging | 59 |
| **PhD-SNP**  http://snps.biofold.org/phd-snp/phd-snp.html | Protein Sequence | Between 0 and 1. (If >0.5 mutation is predicted Disease) | 60 |
| **SIFT**  http://sift.bii.a-star.edu.sg/ | Protein Sequence | Ranges from 0 to 1. The amino acid substitution is predicted damaging is the score is <= 0.05, and tolerated if the score is > 0.05. | 61 |
| **Meta-SNP**  http://snps.biofold.org/meta-snp/ | Protein Sequence | Between 0 and 1. (If >0.5 mutation is predicted Disease) | 62 |
| **PANTHER**  http://www.pantherdb.org/tools/index.jsp | Protein Sequence | Between 0 and 1. (If >0.5 mutation is predicted Disease) | 63 |
| **Mutationassessor**  http://mutationassessor.org/r3/ | protein sequence | Disease-associated variants got FIS score >3.5. | 64 |
| **SNAP**  https://www.rostlab.org/services/SNAP/ | protein sequence | Output normalized between 0 and 1 (If >0.5 mutation is predicted Disease) | 65 |
| **PROVEAN**  http://provean.jcvi.org/ | protein sequence | Variants with a score equal to or below -2.5 are considered "deleterious,"  -Variants with a score above -2.5 are considered "neutral | 66 |
| **SNPs&GO**  http://snps.biofold.org/snps-and-go/snps-and-go.html | Protein Sequence or | Disease probability (if >0.5 mutation is predicted Disease) | 67 |
| **NetDiseaseSNP 1.0**  http://www.cbs.dtu.dk/services/NetDiseaseSNP/ | Protein Sequence | NetDiseaseSNP score: 0 to 1 (score>=0.5: DISEASE; score<0.5: NEUTRAL).NetDiseaseSNP predicted category for the variant: DISEASE/NEUTRAL | 68 |
| **Align GVGD**  http://agvgd.hci.utah.edu/agvgd_input.php | Protein Sequence | Class C65 <=> most likely  Class C0 <=> less likely | 69 |
| **I-Mutant 3.0**  http://gpcr2.biocomp.unibo.it/cgi/predictors/I-Mutant3.0/I-Mutant3.0.cgi | Protein Sequence | DDG<0: Decrease Stability  DDG>0: Increase Stability | 70 |

**Supplementary Table S3. List and information of web server used in this article.**
